# Comparison of the accuracy between guided and freehand placement of periorbital implants—a cadaveric split-face study

**DOI:** 10.1186/s13005-025-00569-8

**Published:** 2025-11-26

**Authors:** Britta M. Lohn, Stefan Raith, Philipp Winnand, Mark Ooms, Tino Rickert, Nina Wagenknecht, Frank Hölzle, Ali Modabber

**Affiliations:** Department of Oral and Maxillofacial Surgery, University Hospital RWTH Aachen, Pauwelsstraße 30, Aachen, D-52074 Germany

**Keywords:** Surgical template, Craniofacial prosthesis, Extraoral implantology, CMF implants, Anaplastology, Epithesis

## Abstract

**Background:**

Orbital exenteration (exenteratio orbitae) is a disfiguring procedure performed with tumor resection. Rapid realization of an implant-retained craniofacial prosthesis with a secure fit for an active life is essential to restore quality of life. Osseointegrated implants are commonly used for maxillofacial rehabilitation. The precise positioning of these implants is more difficult in cases of reduced bone availability, but it enables anaplastologists to achieve an unobtrusive restoration.

**Methods:**

After computed tomography (CT) scans of 13 cadaver heads, 104 craniomaxillofacial (CMF) implants were digitally planned. Using a split-face study design, one periorbital side was treated with customized surgical guides and one side was operated freehand.

The digital evaluation of position, axis, and insertion depth compared to the digital planning was conducted for 78 periorbital implants using digital evaluation of a postoperative CT scan to measure the linear and angular deviation from preoperative planning.

**Results:**

The linear deviation in 3D (*p* = 0.0105), drilling depth (*p* = 0.0013), and angular deviation (*p* = 0.0004) were significantly greater in the freehand group than in the guided computer-assisted implant surgery (CAIS) group.

**Conclusion:**

Digital planning enables the available bone support to be preoperatively estimated. CAIS with surgical guides offers significantly more accurate results for CMF implant placement than a freehand transfer of digital planning. Guided CAIS requires a larger surgical approach and therefore fails to achieve the goal of a minimally invasive technique.

## Introduction

Orbital exenteration (exenteratio orbitae) is performed in cases of tumor, inflammation, congenital defects, or ballistic trauma. It presents a challenge for reconstruction, requiring perfect surgical skills, exact planning, and aesthetic knowledge [5, 7, 30, 33, 41, 43]. Implant-retained craniofacial prosthesis offers a standardized alternative [3, 14, 23, 51] to reconstructive surgery with autologous tissue, especially when patients’ health prevents a complex microvascular transplant [29, 31] or they refuse prolonged treatment until the aesthetic result is achieved. In some cases, an open defect may be indicated for tumor follow-up and early recurrence detection [7, 44, 45].

Bone-anchored implant mechanisms for cranio-maxillofacial (CMF) implants have revolutionized craniofacial prosthetic rehabilitation. The prosthesis is comfortable to wear, easy to clean, and self-align in an anatomically correct position. For physically active people, implant-supported prostheses promise a more secure fit than bonded facial prosthesis [18]. The difficulty lies in sufficient bone supply while protecting anatomical structures, such as the frontal sinus or the infraorbital nerve [28].

In addition, anaplastologists can achieve better aesthetic results when implants are positioned precisely. A sufficient circular bone thickness of at least 1 mm must be ensured for healing without peri-implantitis or the risk of bone/implant loss [1, 6]. Today, the transfer of the favored position at 7, 8, and 11 o’clock, in relation to the patient's right eye, a distance of at least 10 mm, and a slight inward angulation is expected with digital planning [52, 56, 57]. Following irradiation of the orbital region, Baum et al. [7] showed a five-year survival rate of 86% compared to a survival rate of 92% in non-irradiated patients for craniofacial implants [17, 20]. Consequently, an additional implant is often inserted to ensure sufficient stability even in case of implant failure. Thus, it is necessary to obtain suitable methods of reconstruction tailored to each patient [10].

Unlike dental computer-assisted implant surgery (CAIS) where tooth-, mucosa-, or bone-supported guiding promises precise implant positioning along the planned axis and depth [49, 50], there are few reports on guided implant placement for extraoral implants: tooth-supported splints for ear reconstruction, helmets or goggles for nasal reconstruction, or soup-dish-shaped guides for transcutaneous marking in the orbital region with methylene blue, supplying an individual defect, were presented as case reports [10, 36, 49, 50, 52].

Dings et al. [15] achieved an acceptable accuracy for orbital implants using only a soft tissue-supported template. In this ex vivo study, fully guided implant placement was performed after orbital exenteration with significant bone exposure. Similarly, Veit et al. [52] described the planning process for a 3D-printed, soft-tissue-supported guide using CoDiagnostiX™ (DentalWings, Montréal, Canada) in their report on periorbital implantation.

For CMF implants in orbital prostheses, maintaining the correct inter-implant distance and the skin exit point (≥ 10 mm) is far more important for long-term success than strict adherence to parallelism, as magnets, retaining bars and angulated abutments compensate for moderate angular deviations. However, implants that are placed too close together and greater deviations from the permissible retention zone or greater angular deviations impair hygiene, retention, and the inconspicuous fabrication of the prosthesis [52].

To our knowledge, there is no standardized method for reliably transferring digital planning for the placement of periorbital implants, especially when using a new guide design with combined soft tissue and bone support. This study evaluates the accuracy of a new promising guide design, combining a soft-tissue-supported and bone-supported template, compared with a freehand implant placement based on 3D planning in a split-face model.

## Material and methods

### Study design

After institutional approval (ethics committee of the Medical Faculty of RWTH Aachen, Germany [EK219/16]), 13 human body donors, who willed their body to science during their lifetime, were scanned using computed tomography (CT) with 1 mm slices for analysis using a 128-row multidetector CT scanner (Somatom Definition Flash; Siemens Medical Systems, Erlangen, Germany). The tube voltage was set to 120 kV, the pitch was 0.6, and the tube current was modulated according to Siemens CareDose4D. Following conversion to Digital Imaging and Communications in Medicine (DICOM) format, the data were uploaded to CoDiagnostiX™ (DentalWings, Montréal, Canada) for suitable threshold segmentation following surface reconstruction for bone and soft tissue.

A total of 104 implants were digitally planned for 13 freshly frozen cadaver heads (up to 6 weeks after death), each with four periorbital implants. In standardized implants (L: 6 mm; Ø 4 mm; Biocomp Industries, Vught, Netherlands), the virtual implant placement was performed according to the virtually estimated available bone volume and aesthetic requirements.

During planning, two supraorbital implants (supraorbital medial and lateral) and two infraorbital implants (infraorbital lateral and medial) were considered on each side, with one side inserted freehand and the other guided in the split-face model.

### Virtual surgical planning and 3D fabrication of surgical templates

**Fig. 1:**
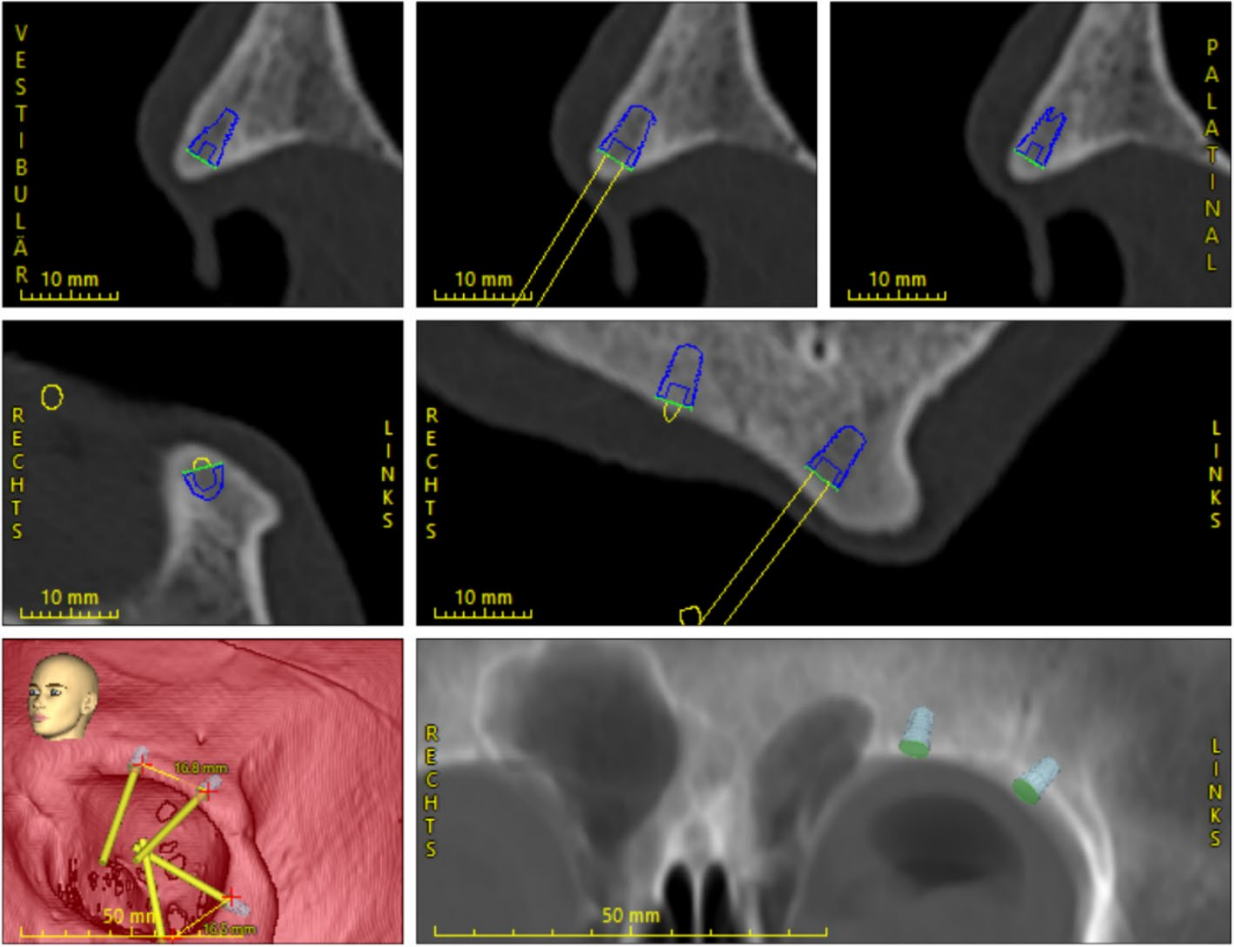
Digital planning in CoDiagnostiX™ (DentalWings, Montréal, Canada) with the imported STL file of the AHEAD implant (L 6 mm; Ø4mm; Biocomp Industries Vught, Netherlands), which show the marked distances between the supra- and infraorbital implants and the implant axis in extension (yellow)

Respecting the prosthetic demands, a maxillofacial surgeon conducted virtual planning. The planning was cross-checked by an experienced oral surgeon with extensive implantological experience in digital planning in CoDiagnostiX™ (DentalWings, Montréal, Canada) (Fig. 1). The geometrical data (STL file format) of the bone-supported templates were designed and exported from CoDiagnostiX™ (DentalWings, Montréal, Canada) and split into supra- and infraorbital rim by the maxillofacial surgeon.

**Fig. 2:**
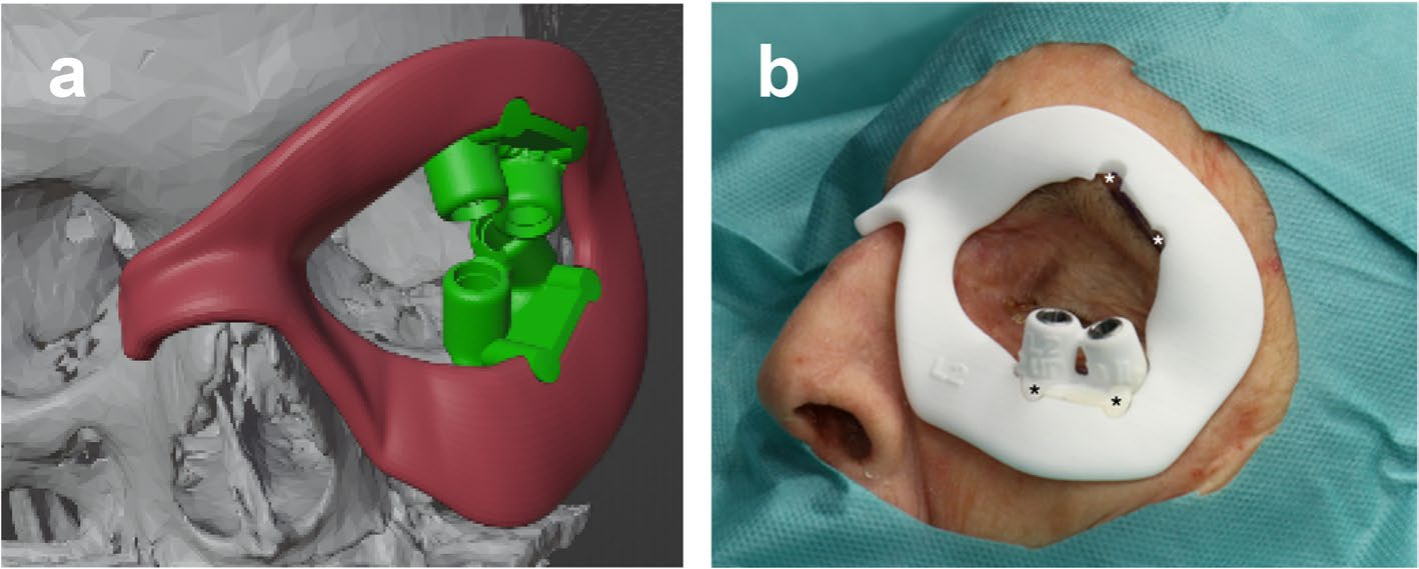
**a** Digital planning using Blender software (version 3.5; Blender Foundation*,* Amsterdam, Netherlands) (green bone-supported template; red soft-supported template). **b** Bone-supported template for the infraorbital rim of the left orbit linked to the soft tissue-supported monocle with extension to the area of the nasion. The supraorbital skin markings in the semicircular anchors also serve as a connection between the bone and the soft tissue element according to the tongue and groove principle

In cooperation with the inhouse surgical mechanical engineer, individual soft tissue frames (red) were designed in the shape of a monocle with extensions around the nasion using the geometrical modeling features of the 3D software Blender (version 3.5; Blender Foundation, Amsterdam, Netherlands). This extension was a positioning aid and protected against rotation and soft tissue displacement. Like dental drilling guides, metallic sleeves were incorporated into the bone-supporting elements (green) of the guide after laser-sintered 3D printing (PROTIQ (Blomberg) EOS P110; PA2200; thickness of 0.10 mm) (Fig. 2a, b).

### Surgical procedure

The freehand group received the digital planning containing marked distances between the supraorbital implants and infraorbital implants and a representation of the implant axis through the digital implant abutments in extension (Fig. 1). In the guided group, the first step was marking the skin at the planned implant position using grooves in the monocle template, which represented the anchor for the bone-supported element.

**Fig. 3:**
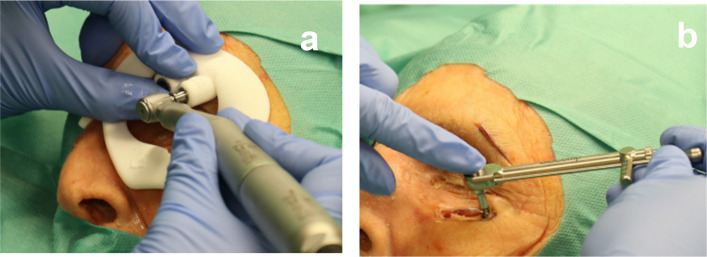
**a** Drilling guided by the drilling log. **b** Implant insertion using a ratchet

Subperiosteal exposure of the bone was followed by insertion of the template and guided drilling according to the AHEAD (Biocomp Industries Vught, Netherlands) drilling protocol (Fig. 3a). After preparing the bony implant site, the implants were placed using the ratchet in accordance with manufacturer instructions to ensure stability. Vertical positioning in the guided group was achieved via the guided drilling stop and in the freehand group via conventional plastic drill sleeves. Implant insertion after drilling was performed without guidance (Fig. 3b). Both procedures were performed by experienced oral and maxillofacial surgeons with routine daily practice in implant planning and insertion. After implant placement, all cadaveric heads were rescanned. The CT data were uploaded and superimposed onto preoperative scans using CoDiagnostiX™ (DentalWings, Montréal, Canada).

### Accuracy analysis

**Fig. 4:**
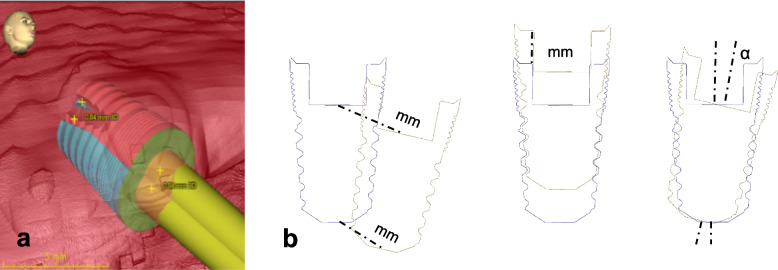
**a** Treatment evaluation using CoDiagnostiX™ (DentalWings, Montréal, Canada) with a semi-automated overlay of the implemented implant planning in the postoperative CT scan (blue) and preoperatively planned position (red). **b** Schematic representation of the angular deviation (in °), the linear deviation in both the entry point and at the apex position, the error of depth insertion at both the implant shoulder and the apex

Using the CoDiagnostiX™ (DentalWings, Montréal, Canada) tool, the semi-automatic superimposition of the planned and resulting implant positions was measured for deviation in the implant tip, shoulder, and axis (Fig. 4).

In addition to the incision length, the soft tissue thickness of the guided group was measured using the digitally determined skin incision and bony implantation points.

In the absence of an objective evaluation system a classification of the accuracy of CAIS (Table 1) was derived to translate evidence-based thresholds from oral implantology [13, 25, 26, 34, 48] into the cranio-maxillofacial epithesis context, accounting for specific clinical tolerances such as angular deviations compensated by magnetic or bar-supported reconstructions [19]. Deviations of less than 2 mm are considered highly accurate [24], while deviations of up to 4 mm or angular deviations of less than 10° remain prosthetically acceptable and are therefore considered clinically adequate (grade I–II). Deviations greater than 4 mm or angular deviations in the double-digit range, on the other hand, cannot be adequately compensated even with anaplastological methods and represent an aesthetic compromise. They are therefore classified as clinically inadequate (grade III–IV), often associated with reconstruction difficulties or implant failure due to critical malpositions:

**Table 1:** Classification of the accuracy of CAIS

Grade	Angular deviation (in °)	3D- offset (in mm)	Comment
Grade I	< 5	< 2	Highly accurate
Grade II	< 10	< 4	Acceptable accuracy
Grade III	< 15	< 8	Inaccurate but prosthetically treatable under compromises
Grade IV	> 15*	> 8*	inaccurate prosthetically not treatable

^*^Including possible damage to anatomical structures/Implant failure

Labeling classified the implantation result as "adequate" or "inadequate" (Table 2).

The labeling of the placed implants for evaluation in inadequate and adequate implant placement with the classification of the accuracy of CAIS (Table 1) resulted in 41 “adequate” implants, with an angular deviation < 10° and a linear deviation < 4 mm compared to 37 “inadequate” implants (Table 2). Of the guided implants, 33.3% were inadequate, and 66.7% were adequate.

**Table 2:** Evaluation of the quality of the implantation: guided vs. freehand

Results	Freehand	Guided	Total
Inadequate	24 (61.5%)	13 (33.3%)	37 (47.4%)
Adequate	15 (38.5%)	26 (66.7%)	41 (52.6%)

Parameters were given in absolute numbers (with percentage). The evaluation was based on the classification of the accuracy of implant placement using digital planning (s. Table 1), whereby an angular deviation up to 10° and a deviation in 3D offset up to 4 mm was declared as adequate for CMF implants

### Statistical analysis

According to the Shapiro–Wilk test, the majority of data did not follow a normal distribution, therefore non-parametric tests were used for analyses. Values were medians with interquartile ranges. Differences between groups were analyzed with the paired samples Wilcoxon test (differences between mirrored implants) or Kruskal–Wallis test (differences between implant positions). The deviation in the insertion depth (apical—base (mm) and apical—tip (mm)) from digital planning was evaluated based on the absolute value. For descriptive analysis, real values were also considered. Associations between parameters were analyzed using the Spearman correlation coefficient (incision length, soft tissue level, and angular deviation). A linear regression analysis was performed of the association between angular deviation and soft tissue thickness, with adjustment for incision length. A similar analysis was conducted on angular deviation and incision length, with adjustments for soft tissue thickness. Significance was assumed when the p value was < 0.05. Analyses were performed using SPSS Statistics (Version 27; IBM Corp.) and GraphPad Prism (Version 10; GraphPad Software Inc.).

## Results

After exposing the bone, a circular cortical bone thickness of at least 1 mm in all direction at the implant shoulder for a standardized implant (Ø4 mm) medially in the supraorbital region was not guaranteed, so actual implant insertion would have been impossible. We focused the analysis exclusively on the standard positions of the lateral supraorbital and medial and lateral infraorbital areas. Consequently, 78 paired implants, 39 guided and 39 freehand, were analyzed.

**Fig. 5:**
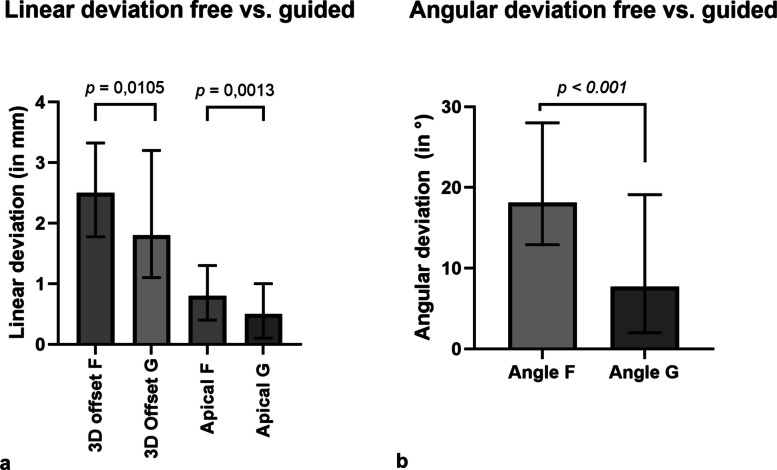
**a** Linear deviation (in mm) and **b** angular deviation (in °) from the virtual plan separately for freehand (F) and guided (G) implant placement. Data are presented as bar columns (median) with interquartile range lines. Differences between groups were analyzed with the paired Wilcoxon-test

Overall, guided implant placement showed significantly higher accuracy than freehand placement for the overall view of linear deviation (*p* = 0.0105) in 3D offset, insertion depth (*p* = 0.0013), and angular deviation (*p* = 0.0004) in digital planning (Fig. 5).

In the freehand group, differences in the insertion depth accuracy depended on the implant position concerning implant depth at both implant shoulder (*p* = 0.021) and implant tip (*p* = 0.006). There was a significant difference between the supraorbital lateral implant positions and the infraorbital lateral (*p* = 0.024) and medial (*p* = 0.011) implant positions. In the guided group, there was no difference between the positions regarding angle, depth, and 3D offset (*p* > 0.05). The insertion depth based on both protocols was insufficient at 57.7% (negative number), whereby the error was greater in freehand group (Table 3). No visual damage to the infraorbital nerve or perforation to the frontal or maxillary sinus was detected.

**Table 3:** Comparison of the insertion depth: guided vs. freehand

Parameters	Freehand	Guided	*p*-value
*Apical – Tip (mm)*	−0.51 (1.2)	−0.01 (1.0)	< 0.001
*Apical – Base (mm)*	−1.06 (1.0)	−0.16 (1.0)	0.02

Parameters are indicated as median values (with interquartile ranges) and separately described for freehand and guided group. Differences between the groups were analyzed using the paired samples Wilcoxon test

The incision length (mm) was significantly greater with guided implantation (freehand in mm): median 23.0 (5.0); guided (in mm): median 32.0 (7.0) (*p* < 0.001). Association analysis was used to analyze the angle deviation and soft tissue thickness (Table 4).

**Table 4:** Association analysis

Variable	B	*p*-value
*Soft tissue thickness (mm)*	0.851 (0.016–1.687)	0.046
*Incision length (mm)*	−1.384 (−2.191–0.578)	0.001

Data presented as regression coefficients (B) (with confidence interval) and p-value according to linear regression analysis (independent variables: soft tissue thickness, incision length; dependent variable: angular deviation)

## Discussion

Computer-assisted surgical planning is indispensable in modern medicine. Studies on oral implants indicate that stable fixation, preferably via occlusion, a simple implantation protocol, and sufficient bone volume can achieve good transferability of the digital planning [21, 22, 42]. Compared to oral implants, extraoral implants face unique challenges. There are fewer cases and often difficult bone and soft tissue conditions, particularly following tumor resections [2, 8–10, 27]. Studies on oral implants show better conditions due to stable attachment points to adjacent teeth and good bone availability or the option for large bone augmentations [12, 48, 55]. In contrast, direct transferability of findings from oral to extraoral implants is limited because of different study designs (in vitro vs. in vivo), type of support (tooth-supported, soft tissue-supported or bony fixation), and anatomical conditions (bone offer, soft tissue cover or unique landmarks) [11, 49, 50].

In this study a split-face model was employed to directly compare guided implant placement and freehand implant placement using the advantage of ex vivo examination. Results indicated that guided placements were more accurate in general than freehand placements both angularly and linearly. Van der Meer et al. [49] inserted two implants in the nasal floor using a tooth-supported guide and achieved remarkable agreement between planned and actual implants, with a deviation of < 2 mm for the implant shoulder and apex and an angular deviation of < 5°. This higher degree of transfer accuracy compared to the results of our study is attributed to the tooth-supported template with a unique fit and good bone quality in the nasal region. However, the accuracy cannot be reproduced or verified due to the small number of cases of only three patients.

Another study by van der Meer et al. [50] using a template for ear reconstruction demonstrated precise transfer accuracy with an average linear deviation of 1.56 mm of the implant shoulder and an average angular deviation of 0.97° of the implant axis in six cadaver heads. The increased accuracy was likely due to reliable fixation points for the stirrup-like guide in the external meatus on both sides, and the bony nasion for ear reconstruction. These are effective designs for precise transfers of defects in the ear and nose area. Conditions in the orbital region are more difficult. Dings et al. [15], examined soft tissue templates with bony screw fixation for periorbital implants in a cadaveric study and obtained a larger mean deviation of up to 2.92 mm around the implant shoulder and an angular deviation of up to 9.39°. Compared to the guided group in this study, a higher transfer accuracy can be achieved with the combined soft tissue-bone-supported guide (median linear deviation of the implant tip freehand: 2.6 mm; guided: 1.8 mm, p-value: 0.018, effect r: 0.377; median angular deviation freehand: 18.1°; guided: 7.7°, p-value: < 0.001, effect r: 0.549) than only the soft tissue-supported guide.

These promising results suggest that accurate positioning through soft tissue support combined with bony support improves implant angulation. Depth safety is crucial for protecting against perforation of the frontal or maxillary sinus and nerve damage during surgery. It is ensured with plastic sleeves for the freehand group and incorporated sleeves in the guided group.

Compared to Soares et al. [35], who described 66.7% of implants as too deep, our study found that fewer implants (43%) were too deep in implants that used plastic or integrated drill stops. Guided implant placement resulted in fewer instances of deep placements, which can protect anatomical structures. In guided or freehand methods, there was no damage to nerves or perforation of frontal or maxillary sinuses. Freehand placements exhibited variability in accuracy, especially concerning insertion depth—supraorbital positions performed worse than infraorbital ones—while guided placements showed consistent results in all positions. This variability arises because freehand techniques rely on visual control, which is more challenging for supraorbital implants. In contrast, guided placements provide uniformity in all positions due to haptic control.

A prerequisite for precise haptic guidance is a unique and correct fit of the guide according to digital planning. In contrast to Dings et al. [15], no exenteration was performed in this study, thus avoiding additional manipulation of soft tissue while accurately determining the incision length required for the CMF implant. After freezing, the defect corresponded to the volume conditions based on established reconstruction procedures (e.g., radial forearm flap or midline forehead flap) [36, 38] due to dehydration and shrinkage on the bulb. However, repeated defrosting of freshly frozen specimens after the preoperative CT scan resulted in unwanted deformation of the cadaver's soft tissue, leading to a loss of stability and consequently tilting and displacement of the guide position [15, 35].

Thus, although the combined bone and soft tissue support design is promising, the guided design provided poor soft tissue support and was limiting due to its bulky intraorbital design. With an average orbital size of 35 mm and a guide height of 13.2 mm for supra- and infraorbital rim, even the insertion of an angled drill is difficult. This limitation may result in deviations. To address this, a C-shaped sleeve could improve insertion and allow visual control, while extending the support to the contralateral periorbital area (goggle-shaped) could increase stability. Alternatively, two separate monocular guides for infraorbital and supraorbital implants would increase precision but also material costs.

Studies on oral implants show that the accuracy of guided implant placement cannot be significantly improved with a deviation of < 2 mm, even if an accuracy of < 0.5 mm is ideal [15, 25, 48]. Due to potential cumulative errors in the process chain (CT, digital planning software, template design, and printing) in addition to the possible deviation of the surgical outcomes from digital planning, a desired safety space of 2 mm to anatomically important structures is required for CAIS in CMF implants [15]. By considering an additional radiation load in the area of the orbit and the brain with a proportional increase in stochastic risk [37], accuracy could be further increased by reducing the layer thickness from 1 mm to 0.5 mm in the CT scan.

The question of whether implants can support a prosthesis is crucial, as it is directly related to adequate versus inadequate outcomes. We established the classification of the accuracy of CAIS to assess the clinical success of CMF implants (grades I–II) based on objective values. When the classification of the accuracy of CAIS was used to evaluate implantation quality, the insertion of guided implants was more effective than freehand insertion in most cases. Due to the individual restoration, angled abutments, and the artistic skills of the anaplastologists, compromised solutions can often replace improper implant placement (grade III) to achieve patient satisfaction [18].

Three periorbital implants are inserted as standard. The insertion of an additional supraorbital medial implant may be considered as a backup if a risk of implant loss exists [20, 32]. However, the narrow tapered cortical bone and the extensive dimensions of the frontal sinus often compromise the selection of the implant angle and position, which are required for adequate circular bone coverage to distribute external forces and achieve sufficient primary stability [16, 39]. Given the circumstances it is difficult to achieve an inconspicuous prosthetic restoration with prominent medial implants [52]. In this study, due to the clinically insufficient bone width in many specimens, the insertion of a supraorbital medial implant after bone exposure was spared.

This study identified the factors influencing angular accuracy. A 1 mm increase in soft tissue thickness resulted in an angular deviation of 0.85°, adjusted for incision length. This was caused by increased mobility due to the greater distance between the soft tissue and bone surfaces, and restricted working space in the orbit. After irradiation, there is consensus regarding the risks of postoperative wound healing disorders, superinfections, and even bone necrosis [17, 18]. To avoid bony exposure associated with bacteremia, a minimally invasive approach or flapless surgery is desirable [4, 47, 53]. In this study, there was no history of radiation therapy in the head and neck area. The effect of irradiated bone on the primary implant outcome potentially requires further study.

To the best of our knowledge, this study is the first to examine incision length and its correlation with transfer accuracy. Contrary to expectations, guided implant placement does not facilitate minimally invasive procedures. Instead, extended cutting length can reduce angular deviation, possibly due to better visibility and secure placement of the bone-supported guide. The following recommendations are made for future practice: To ensure stable guidance, implant systems must be tailored to individual needs (e.g., osseointegrated implants versus subperiosteal fixed titanium plates), and implant sizes based on the available cortical bone width and depth. Particularly supraorbital, selecting a smaller implant diameter because of the narrow cortical bone width offers promising treatment results considering that primary stability is significantly influenced by the cortical bone [16, 39]. Furthermore, drilling should be performed with a drill stop, whether using guided or freehand techniques, to protect anatomical structures. Finally, fully guided implant placement can improve accuracy.

Digital planning is the most reliable step in the digital workflow and promises a reduction in patient visits, operating time, and risks. If digital planning is also transferred to the operating room with sufficient accuracy, digital manufacturing of the prosthesis is also conceivable, which reduces the time required in the laboratory. However, this requires digitally trained and qualified anaplastologists and the acquisition of the appropriate resources [19, 40, 46]. CAIS can be time-consuming and costly but lead to greater accuracy and benefits the patient. A promising future development to reduce inaccuracies in the manufacturing and positioning of the template is digitally navigated, image-guided surgery. The use of supplementary real-time detection offers more precise implant placement and template positioning [9, 54]. Further studies must explore these aspects in relation to CMF implants. This approach could reduce exposure in soft tissue conditions, improve visualization of the stop regardless of implant position, and increase real-time feedback for immediate corrections to the axis, entry point, or drill stop.

With further improvements in clinical implementation in the future, e.g., by adapting the guide or using full navigation, the additional effort is justified. Currently, digital planning for visualizing the bone availability for selecting an individual implant size and estimating soft tissue thickness appears to represent the main added value.

## Conclusion

Digital planning enables the available bone support to be preoperatively estimated. CAIS with surgical guides offers more accurate results for implant placement than a freehand transfer of digital planning. Digitally navigated, image-guided surgery in real time, as used in studies on oral implant placement, can eliminate inaccuracies in the fabrication and positioning of templates. In addition, each patient must be treated individually with a suitable technique.

## Data Availability

No datasets were generated or analysed during the current study.

## Abbreviations

CT: Computed tomography
CMF: Cranio-maxillofacial
DICOM: Digital Imaging and Communications in Medicine
CAIS: Computer-assisted implant surgery
